# Fetoscopic Laser Ablation of Type II Vasa Previa—Case Report and Systematic Review

**DOI:** 10.1002/pd.6897

**Published:** 2025-09-29

**Authors:** Rodrigo Ruano, Olivia Mihulka, Aishu Hombal, Natalia Ravelo, Lara Slesnick, Ugo Maria Pierucci

**Affiliations:** ^1^ University of Miami Miller School of Medicine Miami Florida USA; ^2^ Department of Pediatric Surgery Ospedale Dei Bambini Vittore Buzzi Milan Italy

**Keywords:** fetal surgery, fetoscopic laser ablation, pregnancy prolongation, prenatal diagnosis, vaginal delivery, vasa previa

## Abstract

Vasa previa is a rare but potentially fatal obstetric condition in which fetal vessels traverse the internal cervical os without the protection of placental tissue or Wharton's jelly, making them highly vulnerable to rupture during labor or spontaneous membrane rupture. This can result in rapid fetal exsanguination and death. While current management involves planned cesarean delivery before the onset of labor, fetoscopic laser ablation (FLA) has recently emerged as a minimally invasive alternative for selected cases, particularly type II and III vasa previa, allowing for pregnancy prolongation and potential vaginal delivery. We report the case of a 40‐year‐old gravida 3 para 0 woman diagnosed with type II vasa previa at 22 + 5 weeks. FLA was performed successfully at 31 + 5 weeks without complications. At 36 + 5 weeks, she experienced spontaneous rupture of membranes and delivered a healthy male neonate via cesarean section at maternal request. To contextualize this case, we conducted a systematic review of 54 cases from the literature. FLA was associated with a 98.1% technical success rate, 50% vaginal delivery rate, and 100% neonatal survival, with no major maternal complications reported. These findings suggest that FLA may offer a safe and effective alternative to preterm cesarean delivery in selected patients.

## Highlights

- What is already known about this topic?
  ◦ Vasa previa is associated with a high risk of fetal death due to vessel rupture during labor.
  ◦ Standard management involves elective preterm cesarean delivery to prevent exsanguination.
  ◦ FLA has been proposed as a minimally invasive alternative in selected cases of type II or III vasa previa, but supporting evidence remains limited.
- What does this study add?
  ◦ Demonstrates successful use of FLA at 31 + 5 weeks with favorable maternal and neonatal outcomes.
  ◦ Systematic review of 54 cases revealed a 98.1% technical success rate, 50% vaginal delivery rate, and 100% neonatal survival.
  ◦ Confirms that FLA may safely prolong pregnancy and potentially avoid preterm cesarean section in selected type II/III vasa previa cases.

## 1 Introduction

Vasa previa is a rare but potentially catastrophic obstetric condition characterized by fetal blood vessels traversing the lower uterine segment unprotected by the placenta or Wharton's jelly [1]. These vessels are at risk of rupture during membrane rupture or labor, potentially leading to rapid fetal exsanguination, severe anemia, intrauterine fetal demise (IUFD), or neonatal death. Although improved prenatal imaging has increased the antenatal detection of vasa previa, the condition continues to necessitate cesarean delivery prior to the onset of labor or spontaneous rupture of membranes. Pregnancies complicated by vasa previa are also associated with an elevated risk of preterm birth and maternal hemorrhage [1, 2].

Several placental and cord anomalies have been identified as risk factors for vasa previa, including velamentous cord insertion, bilobed or succenturiate placentas, low‐lying or previa placentas in the second trimester, and pregnancies conceived via assisted reproductive technologies. Vasa previa is categorized into three types: Type I, the most common, involves velamentous cord insertion with fetal vessels coursing over the cervix toward the placental edge. Type II results from bilobed or succenturiate placentas, with fetal vessels connecting the lobes and crossing the internal os. Type III involves abnormal fetal vessels coursing between placental segments without classical features of Type I or II and is often associated with marginal cord insertion or other placental abnormalities [1, 2, 3].

Fetoscopic laser ablation (FLA) has emerged as an innovative intrauterine treatment strategy in selected cases of vasa previa, particularly for Types II and III. This approach aims to coagulate the exposed fetal vessels, potentially allowing prolongation of pregnancy and facilitating vaginal delivery. The first reported successful vaginal birth following FLA for vasa previa was published in 2014 by Johnston et al. [4]. Subsequently, Chmait et al. [5] described a case series of 20 patients with Type II or III vasa previa treated with FLA between 31‐ and 33‐weeks of gestation. In that series, successful vessel occlusion enabled outpatient management, with a mean gestational age at delivery of 37.2 ± 1.8 weeks and a 70% vaginal delivery rate [5]. Despite these promising results, the literature remains limited to a small number of case reports and retrospective series.

In this study, we present a case of successful fetoscopic laser ablation performed at 31 weeks and 5 days of gestation in a patient with Type II vasa previa. The patient was subsequently delivered via cesarean section at 36 weeks and 5 days following spontaneous rupture of membranes, with favorable neonatal outcomes. To contextualize this case within the broader evidence base, we also conducted a systematic review of published cases of FLA for vasa previa. Our objective was to characterize patient selection, procedural success, maternal and neonatal outcomes, and complications associated with this evolving therapeutic approach.

## 2 Methods

**FIGURE 1:**
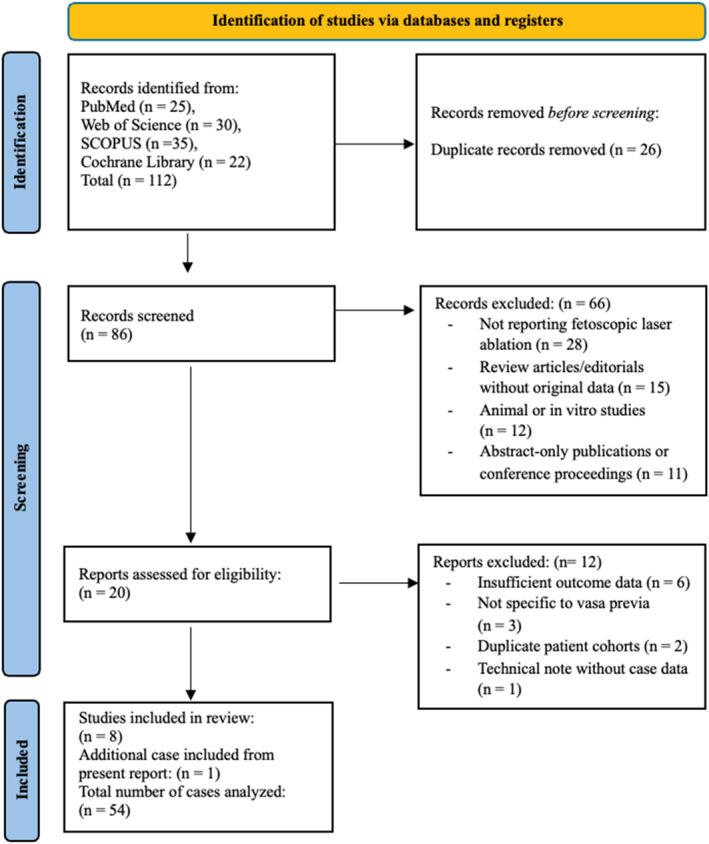
PRISMA flow diagram of study selection—fetoscopic laser ablation for vasa previa.

We report a case of successful fetoscopic laser ablation of type II vasa previa. In addition, we performed a systematic review of the literature. This systematic review was conducted in accordance with the preferred reporting items for systematic reviews and meta‐analyses (PRISMA) guidelines, with the objective of evaluating clinical outcomes associated with FLA for the management of vasa previa. A comprehensive search of the PubMed, Embase, Scopus, and Cochrane Library databases was performed from inception to April 2024, using a combination of keywords and controlled vocabulary, including “fetoscopic laser ablation,” “vasa previa,” “fetal surgery,” and “laser therapy.” No restrictions were applied regarding language, publication year, or geographic location. Studies were considered eligible if they reported original clinical data on pregnant patients with any type of vasa previa who underwent FLA and included outcomes such as gestational age at the time of intervention and delivery, procedural success, latency interval, maternal and neonatal complications, NICU admission, or neonatal survival. Eligible study designs included case reports, case series, cohort studies, and clinical trials. Exclusion criteria comprised studies not involving FLA as an intervention, animal or preclinical studies, and review articles, editorials, or abstracts lacking original patient‐level data. All titles and abstracts were screened independently by two reviewers, with full‐text review conducted for potentially relevant articles; disagreements were resolved through discussion and consensus. Reference lists of the included studies were also manually searched to identify any additional eligible publications. Data were independently extracted using a standardized form and included study design, number of cases, gestational age at FLA and delivery, latency period (defined as days from procedure to delivery), mode of delivery, maternal and neonatal outcomes, and need for repeat intervention. Owing to the small number of reported cases and variability in outcome measures, only qualitative synthesis was performed. Due to the descriptive and case‐based nature of the included studies, no formal risk of bias assessment was performed. The included reports consisted primarily of case reports and small case series, limiting the applicability of standardized risk assessment tools. Descriptive statistics were used to summarize available data, and findings are presented in tabular form (Table 1). The study selection process is depicted in the PRISMA flow diagram (Figure 1).

**TABLE 1:** Summary of published cases of fetoscopic laser ablation for vasa previa (including present case).

Reference	Study type	FLA (*n*)	Successful FLA (*n*)	Success rate (%)	GA at procedure	GA at delivery	Latency days	SVD	NICU admission	Neonatal survival (%)
Chmait et al. (2020) [6]	Retrospective study	10	10	100	28.8 (mean)	35.5 (mean)	48 (mean)	5	0	100
Papanna et al. (2023) [7]	Retrospective study	7	6	85.7	31.1 (median)	35.3 (median)	33 (median)	3	NR	100
Chmait et al. (2024) [5]	Retrospective study	20	20	100	32 (mean)	37.2 (mean)	37 (mean)	14	3	100
Backley et al. (2024) [8]	Retrospective study	11	11	100	NR	35.2 (mean)	NR	4	NR	NR
Chmait et al. (2009) [9]	Case series	2	2	100	29.4 (mean)	34 (mean)	32 (mean)	0	NR	100
Johnston et al. (2014) [4]	Case report	1	1	100	31.7	38.1	38	1	0	100
Hosseinzadeh et al. (2015) [10]	Case report	1	1	100	29.4	34.7	37	0	1	100
Quintero et al. (2007) [11]	Case report	1	1	100	22.7	NR	NR	NR	NR	NR
Present case	Case report	1	1	100	31.7	36.7	36	0	0	100
Total		54	53	98.1				27	4	100

Abbreviations: FLA: Fetoscopic laser ablation, GA: Gestational age, NICU: Neonatal intensive care unit, NR: Not reported, SVD: Spontaneous vaginal delivery.

## 3 Results

### 3.1 Case Report

**FIGURE 2:**
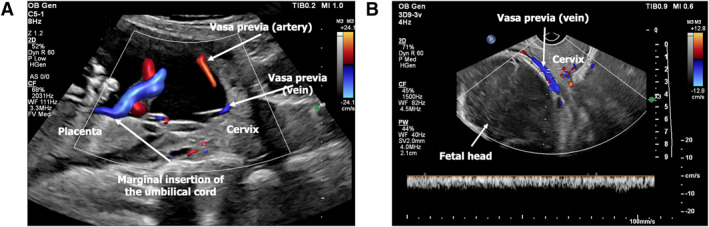
(A) Transabdominal two‐dimensional ultrasonography confirming type II vasa previa: Posterior placenta with marginal insertion of the umbilical cord and two previa vessels (one artery and one vein). (B) Transvaginal ultrasonography confirming the vasa previa.

A 40‐year‐old woman, gravida 3, abortus 2, was referred to our fetal therapy center at 22 weeks and 5 days of gestation due to advanced maternal age and a suspected diagnosis of vasa previa identified on routine obstetric ultrasound. Serial follow‐up ultrasounds confirmed a low‐lying posterior placenta with marginal cord insertion, along with an anterior succenturiate placental lobe. Color Doppler imaging demonstrated a vasa previa composed of one vein and one artery crossing the internal cervical os, consistent with Type II vasa previa (Figure 2A,B).

After comprehensive multidisciplinary counseling regarding the risks and benefits of expectant versus interventional management, the patient elected to undergo fetoscopic laser ablation. She was admitted at 31 weeks and 4 days of gestation for the planned procedure.

**FIGURE 3:**
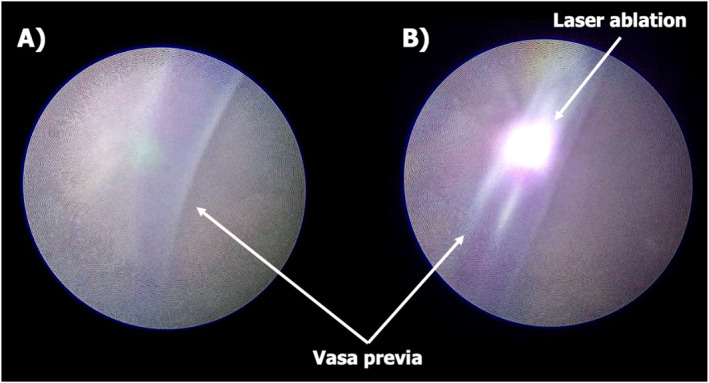
(A) Fetoscopic image of type II vasa previa. (B) Fetoscopic laser ablation of type II vasa previa.

Preoperative management included administration of antenatal corticosteroids to promote fetal lung maturation and initiation of a magnesium sulfate infusion for fetal neuroprotection. Under continuous ultrasound and fetoscopic guidance, the bridging fetal vessels were identified. A 3.3 mm, 0‐degree fetoscope was introduced following amnioinfusion with 1050 mL of warmed saline. Laser ablation of the vessels was performed using diode energy (Figure 3A,B). A total of 9.318 kJ of laser energy was delivered over 5 min and 12 s, for a total of 69 pulses. At the end of the procedure, an ultrasound‐guided amnioreduction was performed to remove the previously instilled amniotic fluid.

**FIGURE 4:**
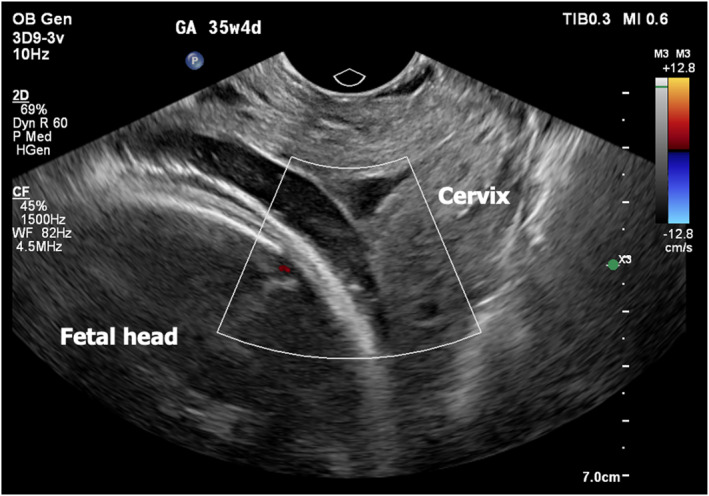
Postsurgical transvaginal ultrasonography showing complete resolution of the type II vasa previa.

Throughout the procedure, the fetal heart rate remained stable, with no evidence of bradycardia. The patient was monitored postoperatively in the labor and delivery unit and completed a 24‐h course of magnesium sulfate. She was subsequently started on oral nifedipine (10 mg every 8 h) for tocolysis. A follow‐up transvaginal ultrasound with Doppler confirmed successful vessel occlusion with no residual vascular flow across the cervix (Figure 4). The postoperative course was uneventful, and the patient was discharged home on postoperative day two.

At 36 weeks and 5 days of gestation, the patient presented with spontaneous rupture of membranes, without uterine contractions. At her request, a primary cesarean section was performed, resulting in the delivery of a male neonate weighing 2415 g, with Apgar scores of 9 at 1, 5, and 10 min. Bilobed placenta was confirmed at delivery. Both the mother and newborn had an uncomplicated postoperative course and were discharged in stable condition.

### 3.2 Systematic Review

The systematic search identified eight eligible studies reporting clinical outcomes following FLA for vasa previa, comprising four retrospective series, one case series, and three case reports, for a total of 53 previously published cases. With the inclusion of our present case, the final analysis encompassed 54 cases (Table 1). Gestational age at the time of FLA ranged from 22 weeks to 5 days to 32 weeks and 5 days. The primary indications for FLA included parental preference for outpatient management, avoidance of preterm cesarean delivery, and potential for vaginal birth. All procedures were performed via fetoscopic access using either transabdominal or transvaginal entry, and laser coagulation was achieved using a 600‐μm or 800‐μm diode laser fiber, similar to techniques employed in twin‐to‐twin transfusion syndrome (TTTS). Technical success, defined as complete vessel occlusion without the need for repeat intervention, was achieved in 98.1% of cases (53/54). One patient required a second procedure due to persistent flow, and one underwent emergency cesarean delivery on the same day due to unsuccessful ablation. The latency interval, defined as the number of days between the FLA procedure and delivery, ranged from 32 to 48 days where reported. Delivery occurred prior to 37 weeks in most cases, although two studies reported deliveries beyond 37 weeks, including a mean gestational age of 37.2 ± 1.8 weeks in the largest series. Vaginal delivery was achieved in 50% of cases (27/54), while the remainder underwent cesarean section. NICU admission was required in four neonates, although this outcome was inconsistently reported across studies. Importantly, the neonatal survival rate was 100% in all included cases, and no maternal complications, such as hemorrhage, chorioamnionitis, or sepsis, were documented. The present case followed a clinical trajectory consistent with prior reports, with successful vessel ablation at 31 + 5 weeks, latency of 36 days, and cesarean delivery at 36 + 5 weeks due to spontaneous rupture of membranes, resulting in favorable maternal and neonatal outcomes.

## 4 Discussion

The standard of care for managing vasa previa involves planned cesarean delivery between 34 and 37 weeks of gestation in order to preempt the catastrophic risk of fetal exsanguination resulting from vessel rupture during labor or spontaneous rupture of membranes [1, 2]. However, this approach may not be feasible in all clinical scenarios, particularly in cases complicated by antenatal bleeding, uterine activity, or unexpected events prior to fetal viability. In such high‐risk situations, FLA has emerged as a potential alternative, offering the possibility of sealing exposed fetal vessels and thereby reducing the risk of acute hemorrhage while enabling pregnancy prolongation and improving perinatal outcomes [4, 5]. Although still considered investigational, FLA has shown promise in a limited number of reports, primarily for Type II and III vasa previa, with some cases achieving term delivery and even vaginal birth following successful intervention [4, 5, 12]. Nevertheless, ongoing concerns persist regarding the risks of preterm premature rupture of membranes (PPROM), technical failure, and vessel recanalization, emphasizing the need for meticulous patient selection and procedural expertise [7, 13].

In the present case, we successfully performed FLA at 31 weeks and 4 days of gestation in a patient with Type II vasa previa. Postoperative transvaginal ultrasonography confirmed no signs of vasa previa anymore. The pregnancy was subsequently prolonged by 36 days, culminating in cesarean delivery at 36 weeks and 5 days following spontaneous rupture of membranes without vaginal bleeding confirming that the vasa previa had been successfully coagulated. Post‐procedurally, the patient was managed in an outpatient setting without complications, and both maternal and neonatal outcomes were favorable. The procedure was conducted under direct fetoscopic and ultrasonographic visualization, enabling precise localization and coagulation of the bridging vessels while minimizing the procedural risk. This outcome underscores the potential of FLA to serve as a safe and effective alternative to prolonged antepartum hospitalization in appropriately selected cases.

In our systematic review of the published cases of FLA for type II vasa previa (*n* = 54), we found that the procedure was almost uniformly successful, with a technical success rate of 98.1%. The gestational age at which FLA was performed ranged from 22 + 5 to 32 + 5 weeks, reflecting both early second‐trimester interventions and more recent third‐trimester attempts. Over time, there has been a trend toward later procedures (i.e., 30–32 weeks), which seem to balance procedural safety and fetal maturity [1]. Indeed, mean latency from FLA to delivery ranged from 32 to 48 days, enabling many fetuses to progress to late‐preterm or even term delivery. Notably, 50% (27/54) of cases culminated in successful vaginal delivery, highlighting a marked departure from the current paradigm of routine preterm cesarean delivery for vasa previa [5, 7, 14]. Neonatal outcomes were consistently favorable, with 100% survival and no periprocedural deaths reported. NICU admission, though inconsistently reported, was generally due to mild prematurity‐related issues. Most notably, no maternal complications such as hemorrhage, infection, or procedure‐related morbidity were documented in any of the reviewed cases. These findings underscore that in experienced hands, FLA is technically feasible, safe, and potentially reproducible across centers. However, the total sample size remains small and subject to publication bias, with limited data on long‐term outcomes. Additionally, patient selection criteria, timing of intervention, and operative protocols remain highly variable, reinforcing the need for standardized guidelines.

This case and the systematic review also underscore several key considerations. First, careful patient selection is essential. FLA is best suited for cases of Type II or III vasa previa in which the course of the exposed vessels can be clearly visualized and accessed. Type I case, involving velamentous cord insertion at the placental margin, often pose greater technical challenges. Second, optimal timing of the procedure must balance the risk of spontaneous rupture against the goal of advancing gestational age to reduce neonatal morbidity. In our case, the intervention was performed at 31 + 4 weeks, approaching the gestational threshold of 32 weeks, beyond which neonatal outcomes improve significantly [13]. Notably, Chmait et al. [6] reported that after modifying their institutional protocol to delay FLA until after 31 weeks, the mean gestational age at delivery increased to 38.1 ± 1.4 weeks, suggesting that later intervention may confer enhanced benefit in terms of gestational prolongation [6].

Despite these encouraging findings, several limitations and challenges must be acknowledged. FLA requires a high level of technical expertise, access to specialized equipment, and the availability of a multidisciplinary fetal therapy team, factors that limit its accessibility to highly resourced centers. Postoperative monitoring remains critical, with vigilance for complications such as chorioamnionitis, PPROM, and vessel recanalization. Moreover, while the safety profile appears favorable, our review found no reports of major maternal complications across all documented cases, including our own; the small number of patients and lack of long‐term neonatal follow‐up limit the strength of the conclusions. Importantly, the immediate neonatal survival rate was 100% across the cohort, but the heterogeneity in reporting of NICU admissions, delivery indications, and long‐term neurodevelopmental outcomes precludes definitive safety assessment.

At present, the evidence base supporting FLA for vasa previa is limited to case reports and small series, restricting generalizability and precluding the establishment of standardized protocols. Larger prospective studies and ideally randomized controlled trials are needed to define the true efficacy, safety, and durability of this intervention. Future research should also aim to identify precise selection criteria, determine the optimal gestational window for intervention, and evaluate adjunctive therapies, such as amniopatch application for managing membrane integrity, to further enhance procedural success and reduce associated risks. As fetoscopic techniques continue to advance, FLA may become an increasingly viable option for selected patients with high‐risk vasa previa, representing a shift from a purely expectant paradigm toward proactive, individualized fetal intervention.

## 5 Conclusion

Fetoscopic laser ablation represents a promising investigational approach for the antenatal management of selected cases of Type II and III vasa previa. In this case, FLA enabled safe prolongation of pregnancy without complications, culminating in a favorable neonatal outcome. Our systematic review reinforces the feasibility, high procedural success rate, and reassuring short‐term safety profile of FLA across a limited but growing body of literature. While current evidence remains confined to small series and case reports, the consistent 100% neonatal survival rate and absence of significant maternal complications are encouraging. Further prospective studies are essential to establish standardized indications, optimal timing, and long‐term outcomes, and to define the role of FLA within the broader management algorithm for vasa previa.

## Ethics Statement

The authors are accountable for all aspects of the work in ensuring that questions related to the accuracy or integrity of any part of the study are appropriately investigated and resolved. All procedures performed were conducted in accordance with the ethical standards of the University of Miami/Jackson Memorial Hospital institutional review board (IRB) and with the principles of the Declaration of Helsinki and its amendments. According to institutional policy, formal IRB approval was waived for this retrospective analysis involving de‐identified clinical data from a single patient.

## Consent

Written informed consent for publication of this case and associated images was obtained from the patient. All data collected were handled in compliance with relevant privacy and data protection regulations, including the Health Insurance Portability and Accountability Act (HIPAA).

## Conflicts of Interest

The authors declare no conflicts of interest.

## Supporting information


supporting Information S1

## Data Availability

The data supporting the findings of this study are available from the corresponding author upon reasonable request. All data analyzed in the systematic review are from published sources, which are cited and referenced in the manuscript.
